# Machine learning for the early prediction of infants with electrographic seizures in neonatal hypoxic‐ischemic encephalopathy

**DOI:** 10.1111/epi.17468

**Published:** 2022-12-20

**Authors:** Andreea M. Pavel, John M. O'Toole, Jacopo Proietti, Vicki Livingstone, Subhabrata Mitra, William P. Marnane, Mikael Finder, Eugene M. Dempsey, Deirdre M. Murray, Geraldine B. Boylan, ANSeR Consortium, Elena Pavlidis, Liudmila Kharoshankaya, Sean R. Mathieson, Gordon Lightbody, Jackie O’Leary, Mairead Murray, Jean Conway, Denis Dwyer, Andrey Temko, Taragh Kiely, Anthony C. Ryan, Janet M. Rennie, Linda S. de Vries, Lauren C. Weeke, Mona C. Toet, Johanneke C. Harteman, Mats Blennow, Ingela Edqvist, Adrienne Foran, Raga Mallika Pinnamaneni, Jessica Colby‐Milley, Divyen K. Shah, Nicola Openshaw‐Lawrence, Ronit M. Pressler, Olga Kapellou, Alexander C. van Huffelen

**Affiliations:** ^1^ INFANT Research Centre University College Cork Cork Ireland; ^2^ Department of Paediatrics and Child Health University College Cork Cork Ireland; ^3^ Institute for Women's Health University College London London UK; ^4^ Electrical & Electronic Engineering School of Engineering University College Cork Cork Ireland; ^5^ Department of Neonatal Medicine Karolinska University Hospital Stockholm Sweden; ^6^ Division of Paediatrics, Department CLINTEC Karolinska Institutet Stockholm Sweden

**Keywords:** machine learning, neonatal encephalopathy, neonatal seizures, neonates, prediction algorithm

## Abstract

**Objective:**

To assess if early clinical and electroencephalography (EEG) features predict later seizure development in infants with hypoxic‐ischemic encephalopathy (HIE).

**Methods:**

Clinical and EEG parameters <12 h of birth from infants with HIE across eight European Neonatal Units were used to develop seizure‐prediction models. Clinical parameters included intrapartum complications, fetal distress, gestational age, delivery mode, gender, birth weight, Apgar scores, assisted ventilation, cord pH, and blood gases. The earliest EEG hour provided a qualitative analysis (discontinuity, amplitude, asymmetry/asynchrony, sleep–wake cycle [SWC]) and a quantitative analysis (power, discontinuity, spectral distribution, inter‐hemispheric connectivity) from full montage and two‐channel amplitude‐integrated EEG (aEEG). Subgroup analysis, only including infants without anti‐seizure medication (ASM) prior to EEG was also performed. Machine‐learning (ML) models (random forest and gradient boosting algorithms) were developed to predict infants who would later develop seizures and assessed using Matthews correlation coefficient (MCC) and area under the receiver‐operating characteristic curve (AUC).

**Results:**

The study included 162 infants with HIE (53 had seizures). Low Apgar, need for ventilation, high lactate, low base excess, absent SWC, low EEG power, and increased EEG discontinuity were associated with seizures. The following predictive models were developed: clinical (MCC 0.368, AUC 0.681), qualitative EEG (MCC 0.467, AUC 0.729), quantitative EEG (MCC 0.473, AUC 0.730), clinical and qualitative EEG (MCC 0.470, AUC 0.721), and clinical and quantitative EEG (MCC 0.513, AUC 0.746). The clinical and qualitative‐EEG model significantly outperformed the clinical model alone (MCC 0.470 vs 0.368, *p*‐value .037). The clinical and quantitative‐EEG model significantly outperformed the clinical model (MCC 0.513 vs 0.368, *p*‐value .012). The clinical and quantitative‐EEG model for infants without ASM (*n* = 131) had MCC 0.588, AUC 0.832. Performance for quantitative aEEG (*n* = 159) was MCC 0.381, AUC 0.696 and clinical and quantitative aEEG was MCC 0.384, AUC 0.720.

**Significance:**

Early EEG background analysis combined with readily available clinical data helped predict infants who were at highest risk of seizures, hours before they occur. Automated quantitative‐EEG analysis was as good as expert analysis for predicting seizures, supporting the use of automated assessment tools for early evaluation of HIE.

**Key points**

- Early electroencephalography (EEG) background combined with clinical information can predict infants which later develop seizure in hypoxic‐ischemic encephalopathy (HIE).
- Automated quantitative‐EEG analysis was as predictive as an expert neurophysiologist analysis for predicting seizures, supporting the use of automated assessment tools.
- The current machine‐learning (ML) prediction models could be used as objective bedside tools to identify which neonates with HIE are at the highest risk of seizures.

## 1 INTRODUCTION

Neonatal encephalopathy caused by perinatal hypoxia‐ischemia is a worldwide health problem and a major cause of mortality and morbidity, with incidence ranging from 1.5–3 per 1000 live births in high‐income countries to 14.9 per 1000 live births in low‐income countries.^1^, ^2^ Brain injury following hypoxic‐ischemic injury evolves over subsequent hours and days and is associated with changing clinical and electroencephalography (EEG) features.^3^, ^4^ Hypoxic‐ischemic encephalopathy (HIE) is the main cause of seizures in full‐term infants and more than half of those with moderate to severe encephalopathy develop seizures.^5^, ^6^ The only treatment currently recommended for moderate and severe HIE is therapeutic hypothermia initiated within 6 h of birth.^7^, ^8^, ^9^ This intervention has been shown to improve long‐term neurodevelopmental outcomes and decrease total seizure burden (TSB).^10^, ^11^ However, even after the introduction of hypothermia, the incidence of seizures in neonates with HIE was estimated by several studies to be between 30% and 65%.^10^, ^12^, ^13^ One study reported that high seizure burden in infants with HIE was associated with poor neurodevelopmental outcome, independent of the severity of encephalopathy.^14^ In addition, regardless of the background etiology, the presence of seizures in neonates is associated with worse neurodevelopmental outcomes and increased mortality.^15^, ^16^

To improve outcomes in this population, prompt recognition and treatment of seizures is vital.^17^, ^18^ However the clinical diagnosis of seizures in neonates is challenging, due to the high frequency of electrographic‐only seizures in HIE and the “uncoupling” phenomenon following anti‐seizure medication (ASM).^19^, ^20^, ^21^ Even when clinical manifestations are present, it can be difficult to differentiate seizures from normal neonatal behaviors.^21^ The gold standard for seizure diagnosis is continuous video‐EEG monitoring, as recommended by the American Clinical Neurophysiology Society (ACNS) and the International League Against Epilepsy (ILAE).^22^, ^23^ Unfortunately, continuous EEG monitoring requires specialized equipment and expert personnel, and is used only for short recordings in most neonatal centers, thereby reducing the likelihood of seizure detection. To overcome this and improve the allocation of scarce resources, a solution would be early identification of infants who are vulnerable to develop seizures, which should also reduce the delays in treatment and lead to better outcomes.^24^

Previous attempts were made to develop seizure prediction models using clinical, biochemical, and EEG markers, alone or in combination.^25^, ^26^, ^27^, ^28^, ^29^, ^30^, ^31^, ^32^, ^33^ A combination of Apgar score at 5 min, pH <7.0 and delivery room intubation,^25^, ^27^ and EEG background analysis with or without other clinical parameters^28^, ^29^, ^30^, ^31^, ^32^ was previously investigated. All previous attempts were hampered by small, heterogenous neonatal populations, or by lack of prospective, detailed, and early EEG monitoring. Traditionally, logistic regression has been used to develop predictive models for newborns with seizures.^30^, ^31^ However, in recent years, machine learning (ML) techniques have allowed for a more robust analysis of complex data, resulting in development of decision support tools for healthcare professionals.^33^, ^34^, ^35^, ^36^

The aim of this study was to use ML techniques to assess the ability of early clinical parameters and EEG background features, to predict those infants with HIE who later develop seizures, using a large multicenter data set. We hypothesized that an ML model combining early clinical data and EEG background features would have the best ability to predict the infants with HIE at risk of seizures.

## 2 MATERIAL AND METHODS

### 2.1 Study setting and participants

This is a secondary data analysis of infants recruited for two prospective, multicenter, cohort studies (ClinicalTrials.gov Identifier: NCT02160171 and NCT02431780) from eight tertiary‐level neonatal intensive care units across Europe (Ireland, United Kingdom, Sweden, The Netherlands).^6^, ^36^ Ethics committee approval from each recruiting site was obtained before recruitment commenced. Both studies included infants born at ≥36 weeks of gestation, requiring continuous conventional EEG (cEEG) monitoring because they were at high risk of developing seizures. For this analysis we included infants with a clinical and electrographic diagnosis of HIE who had cEEG recording within 12 h of birth and before the development of any electrographic seizures. The diagnosis of HIE was based on an antenatal history of a hypoxic‐ischemic event and the presence of encephalopathy on early neurologic examination (most severe modified Sarnat score within 24 h of life), corroborated with EEG background and/or magnetic resonance imaging (MRI) changes consistent with HIE injury. Infants with encephalopathy from other causes, such as sepsis, meningitis, stroke, metabolic, or genetic, or a combined diagnosis, were excluded from the analysis. In the primary studies, an infant was considered to have seizures if experts identified at least one seizure lasting at least 10 s throughout the EEG monitoring. For this study, infants with only very brief seizures (<30 s) and a total seizure burden (TSB) <2 min throughout the EEG monitoring were excluded from the analysis on the basis that generally these seizures would not have been treated. A subgroup analysis was also performed excluding all infants who received ASM before the EEG because of the known effect of ASMs on the background EEG pattern.^37^, ^38^

### 2.2 Early clinical features

The following clinical parameters, available within 6 h of birth, were included in the prediction models: intrapartum complications, suspected fetal distress in labor, gestational age, mode of delivery, gender, birth weight, Apgar scores at 1, 5, and 10 min, assisted ventilation at 10 min of age, lowest cord pH, first postnatal lactate, and first postnatal base excess. Any of the following were considered as an intrapartum complication: placental abruption, ruptured uterus, vasa praevia, intrapartum hemorrhage, cord accident or prolapse, shoulder dystocia, meconium‐stained liquor, or other (poor progression, HELLP syndrome [hemolysis, elevated liver enzymes, low platelet count]/eclampsia, complicated breech presentation, reduced fetal movements, fetal bradycardia, prolonged rupture of membranes).

### 2.3 EEG monitoring and analysis

All infants had cEEG monitoring commencing as soon as possible after birth using NicoletOne ICU Monitor (Natus, USA), Nihon Kohden EEG (Neurofax EEG‐1200, Japan), or XLTek EEG (Natus, USA). Personnel were trained at each study site to position disposable electrodes at F3, F4, C3, C4, Cz, T3, T4, O1/P3, and O2/P4, according to 10:20 EEG electrode neonatal system. The EEG recording had a sampling rate of 250 Hz or 256 Hz, with a filter bandwidth between 0.5 and 70 Hz for review.

For each infant, we extracted the earliest 1‐h epoch of good‐quality EEG recording available before 12 h of age and at least 1 h before the onset of electrographic seizures. All epochs were reviewed, and artifacts were annotated and removed from the quantitative‐EEG analysis. For each EEG epoch included in the analysis, we performed a qualitative (visual) and a quantitative analysis.

The *qualitative‐EEG analysis* was performed by two expert neurophysiologists (GBB and JP) blinded to the seizure status of the infant. The following features were assessed individually as present or absent as per the ACNS^39^: 1, any abnormal discontinuity; 2, discontinuity > 10 s; 3, continuous low voltage to isoelectric; 4, asymmetry and/or asynchrony; 5, sleep‐wake cycles (SWCs) in the first 12 h. Abnormal discontinuity was defined as persistent intervals (interburst intervals [IBIs]) of relatively lower amplitude lasting more than 6 s. Epochs with more marked discontinuity, with IBI >10 s were analyzed separately. A cutoff of 25 μV or less was used to define low voltage, and EEG activity below 2.5 μV in amplitude was referred to as isoelectric activity. Asymmetry was defined as disparity in voltage (more than 2:1) or background feature distribution between homologous areas of the two hemispheres. Asynchrony was defined as noncoherent occurrence of EEG activities (≥1.5 s difference in onset of bursts) over regions on the same or opposite sides of the head. Any differences between neurophysiologists were discussed and a consensus was reached.

Electrographic seizures were defined according to Clancy et al. as at least one EEG channel with sudden, repetitive, and evolving waveforms for a minimum 10 s.^40^ For all infants, electrographic seizures were identified by the same two neurophysiology experts, as described previously.^6^, ^36^ Seizure characteristics were calculated based on these annotations: seizure number, TSB (all seizures during the entire EEG monitoring period, minutes), maximum seizure burden (maximum seizure burden within an hour, minutes/hour), status epilepticus (seizure burden of >30 min within 1 h), seizure period (hours from the beginning of first electrographic seizure to the end of last seizure).

*Quantitative‐EEG analysis* was performed using the NEURAL software package (version 0.4.3), extracting a set of features described previously by our group: power from the 1‐h cEEG epoch, discontinuity, spectral distribution, and inter‐hemispheric connectivity features.^41^ The power features included absolute spectral power and range EEG (median, upper, and lower margin). The discontinuity features included range‐EEG asymmetry, IBI analysis (maximum and median length, percentage, and number of IBIs), amplitude skewness, and kurtosis. IBIs were detected using an algorithm developed for preterm infants^42^ that was also validated in an HIE term cohort.^43^ The spectral distribution features included spectral relative power, spectral flatness, spectral difference, spectral edge frequency, and fractal dimension. Inter‐hemispheric connectivity analysis included connectivity brain symmetry index (BSI) and connectivity coherence. Spectral power (absolute and relative), amplitude skewness and kurtosis, spectral flatness and difference, and coherence and connectivity BSI were generated separately for four frequency bands: 1 to 4 Hz (delta), 4 to 7 Hz (theta), 7 to 13 Hz (alpha), and 13 to 30 Hz (beta). Range EEG is generated for a 1 to 20 Hz band and fractal dimension is generated for 1 to 30 Hz.

Quantitative analysis was also performed using two‐channel EEG from F3‐C3 and F4‐C4. Due to increased usability of two‐channel amplitude integrated amplitude EEG systems (aEEG) in neonatal units, we have developed predictive models separately using quantitative analysis from a reduced montage (F3‐C3 and F4‐C4).

### 2.4 Statistical analysis

Categorical variables were described using frequencies and percentages and continuous variables using means and standard deviations (SDs) when the variables were normally distributed, or medians and interquartile ranges (IQRs) otherwise. For comparisons of continuous variables between two groups (seizure vs nonseizure; moderate HIE vs severe HIE) the independent‐sample *t* test was used for normally distributed variables and the Mann‐Whitney *U* test for non‐normally distributed variables. The chi‐square test or Fisher's exact test (in the case of small, expected counts) was used for categorical variables. All tests were two sided and a *p*‐value <.05 was considered statistically significant. IBM SPSS Statistics (version 24.0, IBM Corp., Armonk, NY, USA) was used for the statistical analysis.

### 2.5 Machine‐learning (ML) analysis

ML models were developed (using Python 3.10.6) separately and in combination for clinical features, and qualitative EEG and quantitative EEG features, to predict infants who later developed seizures. The models were developed using either bagging or boosting ensembles of decision trees. Random forests (a bagging approach) were used to develop models for clinical and qualitative EEG features. Gradient boosting was used for quantitative EEG features. These models were trained and tested using a leave‐one‐out cross‐validation procedure. The depth of the decision tree was optimized from the data. This controlled a level of regularization for the models: A deeper tree captures more information from the feature set but may cause the model to over‐fit. The parameter was selected from a grid search within a nested cross‐validation. This nested scheme employed a 10‐fold cross‐validation repeated 5 times, with each repetition using a different 10‐fold random selection.

#### 2.5.1 Clinical and qualitative EEG features

For the missing clinical data included in the ML model (suspected fetal distress, Apgar scores, assisted ventilation, cord pH, postnatal lactate, and base excess), the mean value of each feature was used for imputation of missing values. Features with more than 50% missing data were excluded. For both clinical and qualitative models, the default number of iterations was used to grow 100 decision trees. The maximum depth of the decision tree was selected in the nested cross‐validation from the set {1,2,3,4}.

#### 2.5.2 Quantitative EEG features

To compensate for the high levels of discontinuous activity in some EEG records, we generated an extra feature set weighted by the quantity of discontinuous activity. This extra feature set consisted of the existing features (excluding the IBI features) multiplied by the percentage of IBIs. We then combined both feature sets for use in building the ML model. ASM status (yes/no if administered before the start of the EEG epoch) and hypothermia status (yes/no if at time of EEG epoch) were also included as adjusting features in the quantitative model. In addition, separate ML models were developed on a subgroup of infants who did not receive any ASM prior to the EEG epoch used in the analysis. We selected the CatBoost implementation of gradient boosting to better manage over‐fitting that can occur with boosting algorithms.^44^ Because this is a small data set for ML (<200 data points) and model variance within the leave‐one‐out strategy can lead to poor performance, we reduced the number of iterations (number of trees) during training from 1000 (default value) to 40 and set the learning rate to 0.1. The depth of the decision tree was selected in the nested cross‐validation from the set {2,3,…,6}.

#### 2.5.3 Assessment and comparison of ML models

To account for the imbalanced classes of seizure and nonseizure, Matthews correlation coefficient (MCC) was used to assess and compare performance of the ML models.^45^ William's test for dependent correlations was used to compare MCCs. More standard metrics were also included: area under the receiver‐operating characteristic curve (AUC), sensitivity, specificity, positive predictive value (PPV), and negative predictive value (NPV). Testing probabilities for the clinical and qualitative EEG models, clinical and quantitative EEG models, and clinical and quantitative aEEG models were then combined using a logit aggregation formula.^46^ For the combined models (clinical and EEG/aEEG models) we used a “late‐stage fusion” method. Agreement between the individual prediction models (clinical, qualitative EEG, quantitative EEG) to quantify for the potential overlap between the models was assessed using Cohen's kappa (values ≤0 indicates no agreement, 0.01–0.20 none to slight, 0.21–0.40 fair, 0.41–0.60 moderate, 0.61–0.80 substantial, and 0.81–1.00 almost perfect agreement).

## 3 RESULTS

From 504 infants recruited in both studies, 266 infants had a diagnosis of HIE, and of those 164 infants had at least 1 h of EEG monitoring before 12 h of age and before emergence of electrographic seizures. Two infants had individual seizures <30 s and a TSB <2 min and were excluded. Hence, 162 neonates with HIE of all grades—mild 62 (38.3%), moderate 69 (42.6%), and severe 31 (19.1%)—were included in the study analysis. Electrographic seizures were present in 53 infants (32.7%) (seizure group). Demographic and clinical characteristics overall and by seizure status group are presented in Table 1. The median (IQR) age at start of the EEG monitoring was 4.7 (3.3–7.7) hours and the median (IQR) age at the time the EEG epoch was analyzed was 6.4 (4.2–8.7) hours, and there were no significant differences in these measures between the seizure and nonseizure groups. Infants with seizures had lower Apgar scores at 1, 5, and 10 min; higher postnatal lactate; and lower base excess; and they were more likely to be cooled and to require assisted ventilation at 10 min of age compared with infants in the nonseizure group. Overall, 31 infants received ASM for clinical concerns of seizures prior to the EEG epoch analyzed.

**TABLE 1:** Study sample demographics and clinical characteristics

	*n*	All infants	*n*	Non‐seizure group	*n*	Seizure group	*p*‐value
				*n* = 109		*n* = 53	
Gestational age at birth (weeks), mean (SD)^a^	162	40.01 (1.32)	109	39.93 (1.29)	53	40.18 (1.37)	.268^b^
Intrapartum complications (yes), *n* (%)^a^	162	140 (86.4)	109	91 (83.5)	53	49 (92.5)	.118^d^
Suspected fetal distress (yes), *n* (%)^a^	145	107 (66)	102	76 (74.5)	43	31 (72.1)	.762^d^
Mode of delivery, *n* (%)^a^							
Unassisted vaginal delivery	162	46 (28.4)	109	30 (27.5)	53	16 (30.2)	.962^d^
Assisted vaginal delivery		62 (38.3)		42 (38.5)		20 (37.7)	
Elective cesarean section		8 (4.9)		5 (4.6)		3 (5.7)	
Emergency cesarean section		46 (28.4)		32 (29.4)		14 (26.4)	
Birth weight (g), mean (SD)^a^	162	3508 (606)	109	3508 (608)	53	3507 (609)	.991^b^
Male gender, *n* (%)^a^	162	103 (63.6)	109	73 (67)	53	30 (56.6)	.198^d^
Apgar at 1 min, median (IQR)^a^	157	2 (1–3)	106	2 (1–3)	51	1 (0–2)	<.001^c^
Apgar at 5 min, median (IQR)^a^	157	4 (2–5)	106	4 (3–6)	51	3 (1–4)	<.001^c^
Apgar at 10 min, median (IQR)^a^	144	5 (4–7)	98	6 (5–8)	46	4 (2–5)	<.001^c^
Assisted ventilation at 10 min (yes), *n* (%)^a^	160	109 (67.3)	108	65 (59.6)	52	44 (83)	.005
Lowest cord pH, mean (SD)^a^	137	7.00 (0.2)	94	7.01 (0.19)	43	6.99 (0.2)	.694^b^
First postnatal base excess, mean (SD)^a^	121	−15.4 (5.8)	85	−14.8 (5.3)	36	−17.1 (6.4)	.037^b^
First postnatal lactate, mean (SD)^a^	102	11.8 (4.32)	69	10.9 (3.9)	33	13.5 (4.7)	.003^b^
HIE grade at discharge							
Mild, *n* (%)	162	62 (38.3)	109	62 (56.9)	53	0	<.001^d^
Moderate, *n* (%)		69 (42.6)		39 (35.8)		30 (56.6)	
Severe, *n* (%)		31 (19.1)		8 (7.3)		23 (43.4)	
Therapeutic hypothermia (yes), *n* (%)	162	133 (82.1)	109	81 (74.3)	53	52 (98.1)	<.001^d^
Age at start of therapeutic hypothermia (h), median (IQR)	133	2 (1–4)	81	2 (1–5)	52	1.5 (1–3)	.070^c^
Age at start of EEG monitoring (h), median (IQR)	162	4.7 (3.3–7.7)	109	5.1 (3.4–7.7)	53	4.2 (2.9–7.1)	.282^c^
Age at start of EEG epoch (h), median (IQR)	162	6.4 (4.2–8.7)	109	6.6 (4.2–8.6)	53	6.1 (4.2–9.5)	.877^c^
Any AEDs given before EEG epoch analyzed (yes), *n* (%)	162	31 (19.1)	109	17 (15.6)	53	14 (26.4)	.101^d^

*Note*: *p*‐value <.05 was considered statistically significant.

Abbreviations: AED, anti‐epileptic drugs; EEG, electroencephalography; HIE, hypoxic‐ischemic encephalopathy; ML, machine learning.

^a^ Variables included in ML model.

^b^ *p*‐Value from independent sample *t* test for parametric data.

^c^ *p*‐Value from Mann‐Whitney test for nonparametric data.

^d^ *p*‐Value from chi‐square test or Fisher's exact test for categorical data.

Electrographic seizure characteristics are presented in Table 2. The median (IQR) age at first electrographic seizure was 14.4 (10.3–19.6) hours. Compared to infants with moderate HIE, infants with severe HIE had a significantly higher number of seizures (median (IQR) 39 (28–104) vs 11^5^, ^6^, ^7^, ^8^, ^9^, ^10^, ^11^, ^12^, ^13^, ^14^, ^15^, ^16^, ^17^, ^18^, ^19^, ^20^, ^21^, ^22^, ^23^, ^24^, ^25^ seizures, *p*‐value <.001), individual seizures were of shorter median duration (94 (68–140) vs 163 (93–838) seconds, *p*‐value .012), TSB was higher (median (IQR) 126 (71–212) vs 59 (26–76) minutes, *p*‐value .003), seizure period was longer (median (IQR) 40.2 (25.3–69.3) vs 9.8 (3.2–32.4) hours, *p*‐value <.001), and they received more ASM (median (IQR) 3^3^, ^4^ vs 1^1^, ^2^ doses, *p*‐value <.001). Eighteen infants (34%) had status epilepticus, and there were no significant differences between the moderate and severe HIE groups.

**TABLE 2:** Seizure characteristics by HIE severity (*n* = 53)

	All infants with seizures	Moderate HIE	Severe HIE	*p*‐value^a^
	*n* = 53	*n* = 30	*n* = 23	
Age at start of EEG monitoring (h), median (IQR)	4.1 (2.9–7.1)	4.3 (3.2–6.6)	4.1 (2.4–7.4)	.566
EEG total monitoring (h), median (IQR)	94.5 (86.4–108.1)	93.6 (86.4–102.6)	95.9 (85.8–120.8)	.44
Age at first electrographic seizure (h), median (IQR)	14.4 (10.3–19.6)	13.5 (8.6–19.4)	14.6 (13.5–19.8)	.229
Number of seizures, median (IQR)	21 (8–51)	11 (5–25)	39 (28–104)	<.001
Median seizure duration (s), median (IQR)	109 (79–355)	163 (93–838)	94 (68–140)	.012
Total seizure burden (min), median (IQR)	71 (33–140)	59 (26–76)	126 (71–212)	.003
Maximum seizure burden (min/h), median (IQR)	23 (15–32)	22 (16–37)	25 (13–32)	.816
Status epilepticus, n (%)	18 (34)	9 (30%)	9 (39.1)	.487^b^
Seizure period (h), median (IQR)	25.3 (8.4–42.7)	9.8 (3.2–32.4)	40.2 (25.3–69.3)	<.001
Received AED treatment at any time (yes), n (%)	49 (92.5)	26 (86.7)	23 (100)	.124^b^
AED before EEG monitoring, n (%)	12 (22.6)	7 (23.3)	5 (21.7)	.891^b^
Total AED doses after EEG start, median (IQR)	2 (1–4)	1 (1–2)	3 (3–4)	<.001

*Note*: *p*‐value <.05 was considered statistically significant.

Abbreviations: HIE, hypoxic‐ischemic encephalopathy; EEG, electroencephalography; AED, anti‐epileptic drugs; IQR, interquartile range.

^a^ *p*‐Value from Mann‐Whitney test for nonparametric data unless otherwise stated.

^b^ *p*‐Value from chi‐square test for categorical data.

EEG features overall and by seizure status group are presented in Table 3 (see Table S1 for all EEG features included in ML models). Low voltage to isoelectric pattern on EEG and absence of SWC were associated with seizure emergence. Several quantitative‐EEG features were significant predictors of seizures. Compared to infants from the non‐seizure group, infants with seizures had significantly lower power (spectral power, for each band and range EEG), lower amplitude kurtosis in the beta band, lower relative power in delta and theta bands, and lower spectral difference in the delta and theta bands. The following features were significantly higher for infants with seizures compared to infants without seizures: range EEG asymmetry, IBI length and percentage, amplitude skewness in delta and theta bands, amplitude kurtosis in the delta band, spectral relative power in alpha and beta band, spectral flatness in beta, spectral difference in the beta band, spectral edge frequency and fractal dimension, connectivity BSI in delta and alpha bands, and mean connectivity coherence in the theta band.

**TABLE 3:** Qualitative and quantitative‐EEG features by seizure status

	Non‐seizure group (*n* = 109)	Seizure group (*n* = 53)	*p*‐value^a^
**Qualitative‐EEG analysis**	***n* (%)**	***n* (%)**	
Discontinuity (yes)	67 (61.5)	26 (50.9)	.134
Discontinuity >10 s (yes)	31 (28.4)	22 (41.5)	.096
Low voltage to isoelectric (yes)	22 (20.2)	36 (67.9)	<.001
Asymmetry and/or asynchrony (yes)	5 (4.6)	3 (5.7)	.767
SWC in the first 12 h (yes)	16 (14.7)	1 (1.9)	.013
**Quantitative‐EEG Features**	**Median (IQR)**	**Median (IQR)**	
*Power features*			
Spectral power delta band	41.8 (19.3–66.9)	5.32 (2.18–29.6)	<.001
Spectral power theta band	8.01 (3.96–11.3)	0.934 (0.427–5.55)	<.001
Spectral power alpha band	3.29 (1.78–4.76)	0.648 (0.316–2.60)	<.001
Spectral power beta band	1.55 (0.95–2.97)	0.727 (0.253–1.75)	<.001
Range EEG lower margin	15.5 (8.20–19.7)	5.61 (4.35–11.4)	<.001
Range EEG median	28.8 (17.3–34.5)	8.94 (6.26–21.5)	<.001
Range EEG upper margin	55.3 (45.2–68.0)	26.0 (14.1–48.7)	<.001
*Discontinuity features*			
Range EEG asymmetry	0.321 (0.277–0.443)	0.471 (0.295–0.644)	.004
IBI length maximum	4.84 (3.27–12.3)	50.9 (8.02–473.0)	<.001
IBI length median	1.88 (1.59–4.12)	16.2 (2.66–48.0)	<.001
IBI percentage	5.37 (1.03–51.6)	90.5 (33.0–98.1)	<.001
Amplitude skewness delta band	0.140 (0.120–0.183)	0.208 (0.135–0.313)	.001
Amplitude skewness theta band	0.011 (0.009–0.017)	0.019 (0.011–0.031)	<.001
Amplitude kurtosis delta band	4.95 (4.44–6.82)	6.22 (4.49–12.1)	.027
Amplitude kurtosis beta band	4.88 (4.04–5.98)	4.20 (3.34–5.03)	.006
*Spectral shape features*			
Spectral relative power delta band	74.9 (70.4–78.9)	69.5 (58.1–76.1)	<.001
Spectral relative power theta band	13.1 (11.0–15.5)	12.0 (9.80–14.0)	.039
Spectral relative power alpha band	6.36 (5.18–7.65)	7.96 (5.58–10.5)	.002
Spectral relative power beta band	3.44 (2.51–5.45)	8.44 (3.59–17.7)	<.001
Spectral flatness beta band	0.757 (0.674–0.814)	0.846 (0.766–0.884)	<.001
Spectral difference delta band	0.010 (0.008–0.013)	0.007 (0.003–0.013)	.009
Spectral difference theta band	0.021 (0.013–0.025)	0.015 (0.006–0.022)	.009
Spectral difference beta band	0.009 (0.006–0.012)	0.011 (0.007–0.015)	.032
Spectral edge frequency	10.4 (8.75–13.0)	17.4 (10.0–22.7)	<.001
Fractal dimension	1.44 (1.40–1.52)	1.65 (1.48–1.76)	<.001
*Connectivity features*			
Connectivity BSI delta band	0.215 (0.173–0.272)	0.257 (0.216–0.324)	<.001
Connectivity BSI alpha band	0.218 (0.185–0.274)	0.254 (0.213–0.327)	.017
Connectivity coherence mean theta band	0.052 (0.040–0.089)	0.072 (0.045–0.154)	.025

*Note*: *p*‐value <.05 was considered statistically significant. Frequency bands: delta: 0.5 to 3 Hz; theta: 4 to 7 Hz; alpha: 8 to 12 Hz; beta: 13 to 30 Hz.

Abbreviations: SWC, sleep‐wake cycle; EEG, electroencephalography; IBI, interburst interval; BSI, brain symmetry index.

^a^ *p*‐Value from Mann‐Whitney test for nonparametric data and chi‐square test for categorical data.

### 3.1 Machine learning (ML) models

Performance of the ML models are presented in Table 4. The MCC (95% CI) for the clinical model was 0.368 (0.219 to 0.506), 0.467 (0.319 to 0.611) for the qualitative‐EEG model, and 0.473 (0.337 to 0.612) for the quantitative‐EEG model. No significance difference was found between the clinical and EEG models: clinical vs qualitative‐EEG models (*p*‐value .083), clinical vs quantitative‐EEG models (*p*‐value .074), and qualitative‐EEG vs quantitative‐EEG models (*p*‐value .425). Cohen's kappa between the clinical model and the quantitative‐EEG model was 0.47 and between the clinical model and quantitative‐EEG model was 0.45. The EEG models (qualitative‐EEG vs quantitative‐EEG models) had a larger Cohen's kappa of 0.89, indicative of the similarity of the models in predicting seizures.

**TABLE 4:** Performance of machine‐learning models (*n* = 162)

ML model	MCC (95% CI)	AUC (95% CI)	Sensitivity	Specificity	PPV	NPV
Clinical model	0.368 (0.219 to 0.506)	0.681 (0.605 to 0.759)	60.4	77.1	56.1	80.0
Qualitative‐EEG model	0.467 (0.319 to 0.611)	0.729 (0.669 to 0.815)	67.9	79.8	62.1	83.7
Quantitative‐EEG model	0.473 (0.337 to 0.612)	0.730 (0.671 to 0.811)	69.8	78.9	61.7	84.3
Clinical and qualitative‐EEG model	0.470 (0.336 to 0.602)	0.721 (0.681 to 0.813)	79.2	70.6	56.8	87.5
Clinical and quantitative‐EEG model	0.513 (0.376 to 0.645)	0.746 (0.700 to 0.833)	75.5	78.0	62.5	86.7

*Note*: Statistical significance between models performance (MCC) using William's test for dependent correlations: clinical vs qualitative‐EEG models, *p*‐value .083; clinical vs quantitative‐EEG models, *p*‐value .074; qualitative‐EEG vs quantitative‐EEG models, *p*‐value .425; clinical and qualitative‐EEG vs qualitative‐EEG models, *p*‐value .475; clinical and qualitative‐EEG vs clinical models, *p*‐value .037; clinical and quantitative‐EEG vs qualitative‐EEG models, *p*‐value .100’ clinical and quantitative‐EEG vs clinical models, *p*‐value .012. Probability threshold was calculated from the receiver‐operating characteristic curve as the point of equal sensitivity and specificity.

Abbreviations: ML, machine learning; MCC, Matthews correlation coefficient; AUC, area under the receiver‐operating characteristic curve; PPV (NPV), positive (negative) predictive value.

Combing the clinical models with the EEG models increased performance: MCC (95% CI) for the combined clinical and qualitative‐EEG model is 0.470 (0.336 to 0.602) and 0.513 (0.376 to 0.645) for the clinical and EEG model. Both models had statistically significant better performances than the clinical model alone: clinical and qualitative‐EEG vs clinical (MCC 0.470 vs 0.368, *p*‐value .037); clinical and quantitative‐EEG vs clinical (MCC 0.513 vs 0.368, *p*‐value .012). MCCs between clinical and qualitative‐EEG vs qualitative‐EEG models (*p*‐value .475) and between clinical and quantitative‐EEG vs quantitative‐EEG models (*p*‐value .100) did not differ significantly.

A calibration curve between the clinical model, the quantitative‐EEG model, and the combined clinical and quantitative‐EEG model is shown as Supplemental Material (Figure S1).

Performances for the ML models using the subgroup of infants with no ASM and aEEG monitoring are presented in Tables 5 and 6, respectively. The aEEG models included 159 infants (due to artifacts on the raw aEEG channels we could not produce an output from 3 infants) and no ASM subgroup included 131 infants (31 infants received at least one dose of ASM before the EEG epoch analyzed). The quantitative‐aEEG model (MCC 0.381) and the clinical and quantitative‐aEEG model (MCC 0.384) had similar performance. For the subgroup of infants without ASM, the clinical and qualitative‐EEG model had an MCC of 0.588.

**TABLE 5:** Subgroup analysis of infants with no ASM given prior to the EEG epoch^a^ (*n* = 131)

ML model	MCC	AUC	Sensitivity	Specificity	PPV	NPV
Clinical model	0.461	0.732	64.1	82.6	61	84.4
Qualitative‐EEG model	0.504	0.706	64.1	85.9	65.8	84.9
Quantitative‐EEG model	0.475	0.806	71.8	78.3	58.3	86.7
Clinical and qualitative‐EEG model	0.519	0.782	76.9	78.3	60	88.9
Clinical and quantitative‐EEG model	0.588	0.832	66.7	90.2	74.3	86.5

Abbreviations: ML, machine learning; MCC, Matthews correlation coefficient; AUC, area under the receiver‐operating characteristic curve; PPV (NPV), positive (negative) predictive value.

^a^ Subgroup analysis of 131 infants (excluding 31 infants who received ASM before the EEG epoch analyzed).

**TABLE 6:** ML models using aEEG^a^ monitoring (*n* = 159)

ML model	MCC	AUC	Sensitivity	Specificity	PPV	NPV
Quantitative‐aEEG model	0.381	0.696	71.2	69.2	52.9	83.1
Clinical and quantitative‐aEEG model	0.384	0.720	61.5	77.6	57.1	80.6

Abbreviations: ML, machine learning; MCC, Matthews correlation coefficient; AUC, area under the receiver‐operating characteristic curve; PPV (NPV), positive (negative) predictive value.

^a^ aEEG = two‐channel EEG (F3‐C3 and F4‐C4) analysis included 159 infants.

## 4 DISCUSSION

In this large multicenter cohort of infants with HIE, 53 of 162 infants developed electrographic seizures at a median age of 14.4 h. Using ML we investigated the ability of the early EEG (recorded at a median age of 6.4 h) and relevant clinical data to predict which neonates would later develop electrographic seizures. Several individual clinical parameters were associated with occurrence of seizures in HIE: low Apgar scores, need for resuscitation at birth, low base deficit, and high lactate. Visual EEG analysis revealed that low voltage and absence of SWC were predictive of seizures in addition to specific power, discontinuity, and spectral shape features on quantitative analysis. Of the individual ML models developed, the EEG models had similar performance (MCCs of 0.467 for qualitative and 0.473 for quantitative), and both outperformed the clinical model alone (MCC 0.368), although these differences were not statistically significant. However, the combined clinical and qualitative‐EEG model significantly outperformed the quantitative‐EEG model alone and the clinical and qualitative‐EEG model significantly outperformed the clinical model alone. Of interest, although the aEEG model did not perform as well as conventional EEG models, performance improved from the clinical model alone, highlighting the value of any EEG monitoring. The combined clinical and quantitative‐EEG model developed exclusively from infants with no prior ASM (*n* = 131) had the best performance (MCC 0.588).

Several previous studies have investigated the predictive value of clinical and EEG parameters for seizure occurrence in neonates. Apgar scores, need for respiratory support, and degree of metabolic acidosis were not found to be reliable predictors of seizures in neonates with HIE.^25^, ^27^, ^28^ Our clinical model included antenatal and postnatal clinical information available within 6 h of birth (the window to start therapeutic hypothermia). The performance of our clinical model (AUC 0.681) was very close to the model reported previously by Sansevere et al.^32^ (AUC 0.662) and outperformed the model reported by Jain et al.^30^ In addition, we have shown that these models work equally well in the first hours after birth when important therapeutic decisions need to be made.

Given the increasing use of EEG monitoring in neonatal units worldwide, EEG background features have also been investigated to assess their ability to predict seizures and outcome and have been shown to outperform clinical parameters. Early abnormal EEG background (discontinuity and low voltage features) has been associated with seizure development.^28^, ^32^, ^47^, ^48^ Consistent with the literature, in our study, early low voltage to isoelectric EEG background was more frequently present in the seizure group compared to the non‐seizure group (67.9% vs 20.2%, *p*‐value <.001). Early SWC was absent in almost all infants with seizures (in our cohort only one infant with later seizures developed SWC before 12 h). In the non‐seizure group, 16 infants had some evidence of early SWC, and all except one were diagnosed with mild encephalopathy. These findings might suggest that when early SWC is present, seizure occurrence is unlikely in the setting of mild HIE, and this could be reassuring for the bedside neonatologist. Quantitative‐EEG analysis is an objective and reproducible analysis of the EEG background activity that does not require expert interpretation by a neurophysiologist. Low EEG power has been correlated previously with a poor neurodevelopmental outcome in neonates.^49^, ^50^ A more recent study has demonstrated that total EEG power < 10 μV^2^ had a specificity of 98%, a sensitivity of 50%, and a PPV of 90% for seizure prediction.^30^ We developed a seizure prediction model for infants with HIE, using a more extensive quantitative‐EEG analysis including several power, discontinuity, spectral distribution, and inter‐hemispheric connectivity features (sensitivity 69.8%, specificity 78.9%, PPV 61.7%, NPV 84.3%). A combination of all these features into a quantitative‐EEG model had better prediction compared to the clinical model and a prediction similar to that of the qualitative‐EEG model (the neurophysiologist's assessment). These findings suggest that quantitative‐EEG analyses can be as accurate as expert interpretation to predict the likelihood of seizures and could augment the neurophysiology service, especially during out of hours periods, to guide the frequency of remote reviewing.

Sansevere et al. developed a combined neonatal seizure prediction model, including clinical information and qualitative‐EEG background features, with an AUC (95% confidence interval [CI]) 0.830 (0.776–0.884).^31^ In comparison, we developed different combined prediction models: clinical and qualitative‐EEG model (AUC (95% CI) 0.721 (0.681 to 0.813)) and clinical and quantitative‐EEG model (AUC (95% CI) 0.746 (0.700 to 0.833)). The addition of clinical parameters to qualitative‐EEG and to quantitative‐EEG analyses significantly improved the predictive ability of clinical model alone. These results emphasize the value of combining clinical and EEG analysis. However, a direct comparison with Sansevere's model is difficult because of several differences between the two studies: We used a more homogeneous population of term infants with HIE; timing of the EEG epochs included in our analysis was standardized to before 12 h of age and before the emergence of electrographic seizures; qualitive analysis was based on the selected epoch and performed by an expert in neonatal EEG blinded to seizure occurrence; the proposed models were tested using a leave‐one‐baby‐out cross‐validation strategy, an unbiased estimator of the generalization performance. This testing process inherently incorporates variance of the models, a likely problem for small data sets such as this, which again minimizes potentially inflated estimates of performance when training and testing with the same data set.

Because many neonatal units worldwide use aEEG monitoring, we also developed a seizure‐prediction model using two‐channel EEG (aEEG – F3‐C3 and F4‐C4).^51^, ^52^, ^53^ Although, the quantitative‐aEEG model was less predictive than the multichannel EEG models, performance improved compared to the clinical model alone. Similarly, adding clinical information to the quantitative‐aEEG analysis improved performance slightly.

The effect of ASM on EEG background was evident in this study by the difference in performance when excluding all infants treated with ASMs prior to the EEG, resulting in models with a superior performance (clinical and quantitative‐EEG model MCC 0.588). However, in clinical practice it would be difficult to exclude these infants, as clinical seizures are often treated before the EEG monitoring is started, especially for those infants that are outborn. Due to the relatively small sample size for this subgroup analysis (*n* = 131 infants), more studies are warranted to account adequately for the effect of ASM.

Several limitations should be considered when interpreting these results. Although this study used a large study sample of infants with HIE, the numbers are small for ML analysis (<200 data points), limiting the ability to build complex ML models and potential bias toward overfitting. However, cross validation can limit this bias in performance assessment. In addition, there are different ways to combine ML models. The method chosen for this study was a late‐stage fusion method, as we found this to be a better strategy than early‐stage fusion for including all features (clinical + EEG) into one model. Due to missing data for blood gas analyses (ranging from 15%–37%), we used mean values to account for this, and included these parameters in the models, even if previous studies have demonstrated no significant change in the predictive value by adding them.^27^ The presence of artifacts in the EEG trace is a limitation when performing quantitative analysis. However, for this analysis we used the first good quality hour of EEG recording, each hour was visually inspected, and artifacts were annotated and removed from the analysis. Unlike detecting seizure events, we are uncertain as to the specific EEG patterns that might be associated with the later development of seizures, other than low power, which is non‐specific. Future work should focus on looking for specific patterns, waveforms, and features in the EEG background associated with later development of seizures. The confounding effect on the EEG background of therapeutic hypothermia and ASM administration in our analysis was reduced by adjusting the quantitative‐EEG model analysis. The presented models were developed for term and near‐term newborns with HIE (36–44 weeks of gestation); therefore new ML models would need to be developed to investigate the likelihood of seizures in other subgroups of newborn infants.

Despite these limitations, this is the first study to use a large cohort of infants with HIE from multiple European neonatal units with early, conventional multichannel EEG monitoring to predict the later development of seizures. The predictive performance of these models included a homogeneous cohort of infants with HIE, as this group represents the main cause of seizures in term newborns and the main reason for EEG monitoring in neonatal units.^5^, ^6^ The EEG background visual analysis and seizure detection were performed by experts in neonatal EEG interpretation, giving more strength to the qualitative‐EEG analysis. EEG analysis was based on an eight‐channel EEG montage, which allowed us to assess cerebral activity across all cortical regions and to detect seizures that might be missed with more limited aEEG monitoring. In addition, a comprehensive quantitative‐EEG analysis was included in the ML analysis as an objective and reproducible quantification of the cerebral activity. Predictive models were developed separately using the eight‐channel EEG and two‐channel EEG aEEG. These models are research focused and will therefore need real‐time clinical validation.

## 5 CONCLUSION

To summarize, early qualitative and quantitative‐EEG features alone can predict infants who will later develop seizures in HIE, hours before seizure onset. Adding available clinical information augments the predictive value of these models. We demonstrated that ML analysis using quantitative EEG is as reliable as an expert (qualitative‐EEG analysis) in predicting the likelihood of seizures. Automated EEG analysis may be useful to individualize the neurophysiology review frequency of continuous EEG monitoring, which may have a significant impact for units with less neurophysiology support. This study opens the door for the implementation of automated ML seizure‐prediction algorithms, as objective bedside tools to identify which neonates with HIE are at highest risk of seizures.

## AUTHOR CONTRIBUTIONS

Andreea M. Pavel recruited participants and collected data, analyzed the data, drafted the initial manuscript, and revised the manuscript. John M O'Toole carried out the quantitative‐EEG analysis and the ML modeling analysis. Jacopo Proietti carried out the qualitative‐EEG analysis. Vicki Livingstone, Subhabrata Mitra, Eugene M. Dempsey, and Deirdre M. Murray were involved in conceptualization and design of the studies and coordinated and supervised data collection and analysis. Geraldine B. Boylan conceptualized, designed, and coordinated both studies; designed the EEG analysis protocol; and carried out the qualitative‐EEG analysis. All authors reviewed and revised the manuscript.

## FUNDING INFORMATION

This study was supported by: A Strategic Translational Award and an Innovator Award from the Wellcome Trust (098983 & 209325); Science Foundation Ireland (15/RI/3239 and 15/SIRG/3580); and the Health Research Board, Ireland (NEPTuNE CDA‐2018‐008). Funders had no role in the design and conduct of the study or in this analysis.

## CONFLICT OF INTEREST

All authors have no conflict of interest to disclose.

## Supporting information


Table S1

Figure S1
