# Supplementary material for: Machine learning for the early prediction of infants with electrographic seizures in neonatal hypoxic‐ischemic encephalopathy
Source: Epilepsia. 2022 Dec 20;64(2):456–68. doi: 10.1111/epi.17468 (PMC10107538; doi:10.1111/epi.17468)
Supplement: Supplementary file 1 — Table S1 [file EPI-64-456-s002.docx]

**Table S1. Complete descriptive analysis for qualitative and quantitative-EEG features**

|  | n | Non-seizure group | n | Seizure group | p-value* |
| --- | --- | --- | --- | --- | --- |
| **Qualitative-EEG analysis** |  | n (%) |  | n (%) |  |
| Discontinuity (yes) | 109 | 67 (61.5) | 53 | 26 (50.9) | 0.134 |
| Discontinuity > 10 sec (yes) | 109 | 31 (28.4) | 53 | 22 (41.5) | 0.096 |
| Low voltage to isoelectric (yes) | 109 | 22 (20.2) | 53 | 36 (67.9) | <0.001 |
| Asymmetry and/or asynchrony (yes) | 109 | 5 (4.6) | 53 | 3 (5.7) | 0.767 |
| SWC in the first 12 hours (yes) | 109 | 16 (14.7) | 53 | 1 (1.9) | 0.013 |
| **Quantitative-EEG Features** |  | median (IQR) |  | median (IQR) |  |
| **Power features** |  |  |  |  |  |
| Spectral power delta band | 109 | 41.8 (19.3 - 66.9) | 53 | 5.32 (2.18 - 29.6) | <0.001 |
| Spectral power theta band | 109 | 8.01 (3.96 - 11.3) | 53 | 0.934 (0.427 - 5.55) | <0.001 |
| Spectral power alpha band | 109 | 3.29 (1.78 - 4.76) | 53 | 0.648 (0.316 - 2.60) | <0.001 |
| Spectral power beta band | 109 | 1.55 (0.95 - 2.97) | 53 | 0.727 (0.253 - 1.75) | <0.001 |
| Range EEG lower margin | 109 | 15.5 (8.20 - 19.7) | 53 | 5.61 (4.35 - 11.4) | <0.001 |
| Range EEG median | 109 | 28.8 (17.3 - 34.5) | 53 | 8.94 (6.26 - 21.5) | <0.001 |
| Range EEG upper margin | 109 | 55.3 (45.2 - 68.0) | 53 | 26.0 (14.1 - 48.7) | <0.001 |
| **Discontinuity features** |  |  |  |  |  |
| Range EEG asymmetry | 109 | 0.321 (0.277 - 0.443) | 53 | 0.471 (0.295 - 0.644) | 0.004 |
| IBI length maximum | 109 | 4.84 (3.27 - 12.3) | 53 | 50.9 (8.02 - 473.0) | <0.001 |
| IBI length median | 109 | 1.88 (1.59 - 4.12) | 53 | 16.2 (2.66 - 48.0) | <0.001 |
| IBI percentage | 109 | 5.37 (1.03 - 51.6) | 53 | 90.5 (33.0 - 98.1) | <0.001 |
| IBI number | 109 | 64.0 (22.3 - 192.3) | 53 | 83 (16.3 - 194.8) | 0.973 |
| Amplitude skewness delta band | 109 | 0.140 (0.120 - 0.183) | 53 | 0.208 (0.135 - 0.313) | 0.001 |
| Amplitude skewness theta band | 109 | 0.011 (0.009 - 0.017) | 53 | 0.019 (0.011 - 0.031) | <0.001 |
| Amplitude skewness alpha band | 109 | 0.010 (0.008 - 0.015) | 53 | 0.011 (0.008 - 0.020) | 0.523 |
| Amplitude skewness beta band | 109 | 0.044 (0.037 - 0.053) | 53 | 0.041 (0.033 - 0.059) | 0.547 |
| Amplitude kurtosis delta band | 109 | 4.95 (4.44 - 6.82) | 53 | 6.22 (4.49 - 12.1) | 0.027 |
| Amplitude kurtosis theta band | 109 | 5.55 (4.66 - 7.45) | 53 | 6.82 (4.40 - 12.0) | 0.174 |
| Amplitude kurtosis alpha band | 109 | 5.17 (4.44 - 6.60) | 53 | 5.05 (3.91 - 8.9) | 0.614 |
| Amplitude kurtosis beta band | 109 | 4.88 (4.04 - 5.98) | 53 | 4.20 (3.34 - 5.03) | 0.006 |
| **Spectral shape features** |  |  |  |  |  |
| Spectral relative power delta band | 109 | 74.9 (70.4 - 78.9) | 53 | 69.5 (58.1 - 76.1) | <0.001 |
| Spectral relative power theta band | 109 | 13.1 (11.0 - 15.5) | 53 | 12.0 (9.80 - 14.0) | 0.039 |
| Spectral relative power alpha band | 109 | 6.36 (5.18 - 7.65) | 53 | 7.96 (5.58 - 10.5) | 0.002 |
| Spectral relative power beta band | 109 | 3.44 (2.51 - 5.45) | 53 | 8.44 (3.59 - 17.7) | <0.001 |
| Spectral flatness delta band | 109 | 0.691 (0.643 - 0.763) | 53 | 0.719 (0.646 - 0.786) | 0.186 |
| Spectral flatness theta band | 109 | 0.867 (0.845 - 0.894) | 53 | 0.857 (0.829 - 0.899) | 0.526 |
| Spectral flatness alpha band | 109 | 0.883 (0.842 - 0.902) | 53 | 0.895 (0.852 - 0.917) | 0.063 |
| Spectral flatness beta band | 109 | 0.757 (0.674 - 0.814) | 53 | 0.846 (0.766 - 0.884) | <0.001 |
| Spectral difference delta band | 109 | 0.010 (0.008 - 0.013) | 53 | 0.007 (0.003 - 0.013) | 0.009 |
| Spectral difference theta band | 109 | 0.021 (0.013 - 0.025) | 53 | 0.015 (0.006 - 0.022) | 0.009 |
| Spectral difference alpha band | 109 | 0.017 (0.013 - 0.021) | 53 | 0.016 (0.007 - 0.021) | 0.296 |
| Spectral difference beta band | 109 | 0.009 (0.006 - 0.012) | 53 | 0.011 (0.007 - 0.015) | 0.032 |
| Spectral edge frequency | 109 | 10.4 (8.75 - 13.0) | 53 | 17.4 (10.0 - 22.7) | <0.001 |
| Fractal dimension | 109 | 1.44 (1.40 - 1.52) | 53 | 1.65 (1.48 - 1.76) | <0.001 |
| **Connectivity features** |  |  |  |  |  |
| Connectivity BSI delta band | 109 | 0.215 (0.173 - 0.272) | 53 | 0.257 (0.216 - 0.324) | <0.001 |
| Connectivity BSI theta band | 109 | 0.220 (0.183 - 0.280) | 53 | 0.241 (0.208 - 0.298) | 0.068 |
| Connectivity BSI alpha band | 109 | 0.218 (0.185 - 0.274) | 53 | 0.254 (0.213 - 0.327) | 0.017 |
| Connectivity BSI beta band | 109 | 0.221 (0.191 - 0.297) | 53 | 0.238 (0.197 - 0.375) | 0.184 |
| Connectivity coherence mean delta band | 109 | 0.119 (0.081 - 0.182) | 53 | 0.112 (0.063 - 0.208) | 0.815 |
| Connectivity coherence mean theta band | 109 | 0.052 (0.040 - 0.089) | 53 | 0.072 (0.045 - 0.154) | 0.025 |
| Connectivity coherence mean alpha band | 109 | 0.050 (0.040 - 0.075) | 53 | 0.059 (0.046 - 0.102) | 0.068 |
| Connectivity coherence mean beta band | 109 | 0.047 (0.036 - 0.074) | 53 | 0.065 (0.037 - 0.089) | 0.094 |

SWC, sleep wake cycle; EEG, electroencephalogram; Frequency bands: delta: 0.5 to 3 Hz; theta: 4 to 7 Hz; alpha: 8 to 12 Hz; beta: 13 to 30 Hz; IBI, inter-burst interval; BSI, brain symmetry index; p-value <0.05 was considered statistically significant; *p-value from independent t-test for parametric data, Mann-Whitney test for nonparametric data and Chi-squared test for categorical data.
