# Supplementary figures and images for: Machine learning for the early prediction of infants with electrographic seizures in neonatal hypoxic‐ischemic encephalopathy
Source: Epilepsia. 2022 Dec 20;64(2):456–68. doi: 10.1111/epi.17468 (PMC10107538; doi:10.1111/epi.17468)

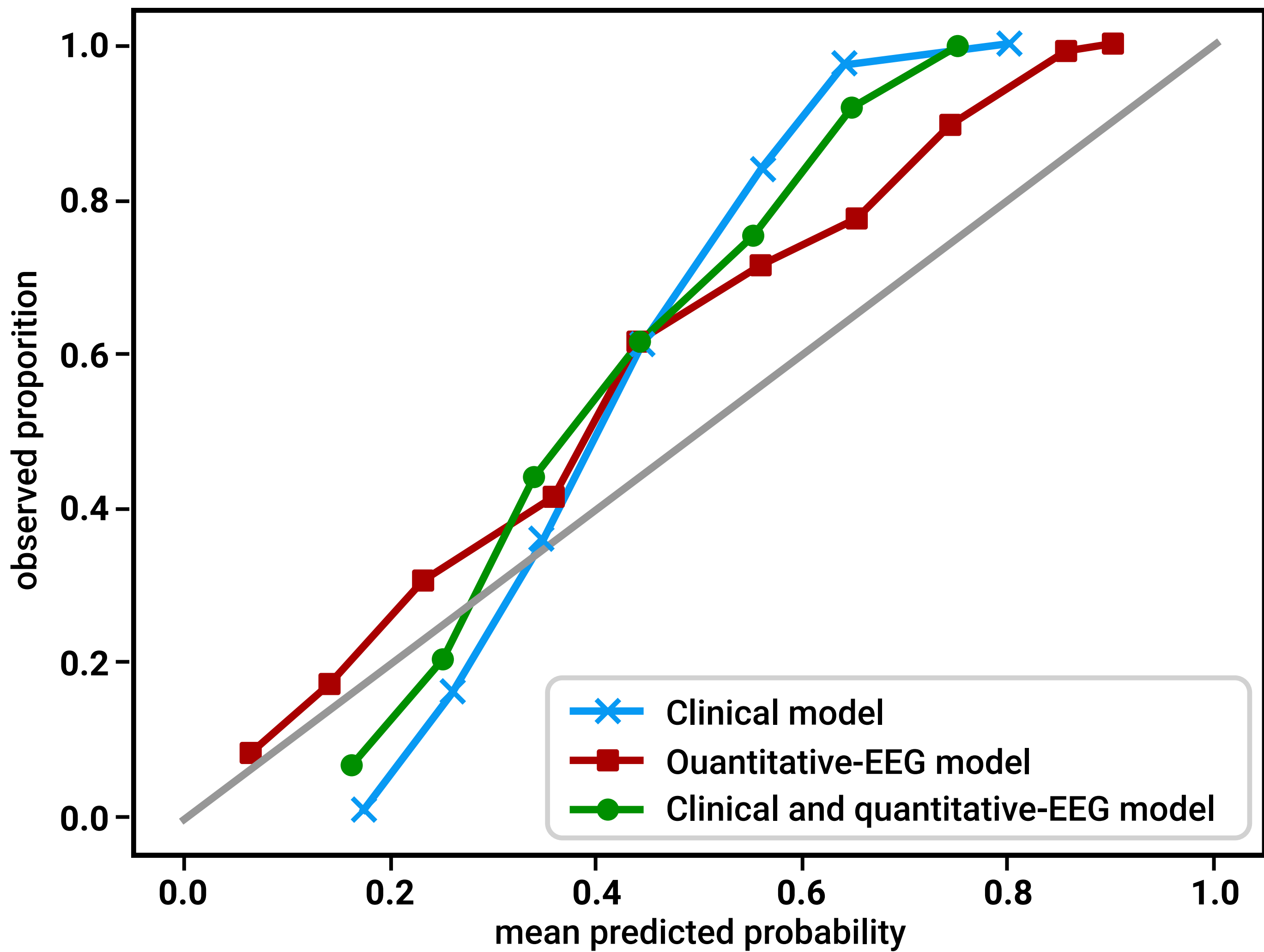

Supplement: Supplementary file 2 — Figure S1 [file EPI-64-456-s001.pdf]
